# 2-(1*H*-Benzimidazol-2-yl)-4-nitro­phenol

**DOI:** 10.1107/S1600536811001644

**Published:** 2011-01-15

**Authors:** Jingya Sun, Xiangdi Tong

**Affiliations:** a College of Marine Sciences, Zhejiang Ocean University, Zhoushan 316000, People’s Republic of China

## Abstract

The title compound, C_13_H_9_N_3_O_3_, was prepared by the reaction of 5-nitro­salicyl­aldehyde with 1,2-diamino­benzene in methanol. The whole mol­ecule is approximately planar, with a mean deviation from the plane defined by the non-H atoms of 0.0311 (4) Å, and with a dihedral angle between the benzene ring and the benzimidazole ring system of 1.1 (3)°. An intra­molecular O—H⋯N hydrogen bond occurs. In the crystal, adjacent mol­ecules are linked through inter­molecular N—H⋯O hydrogen bonds, forming centrosymmetric dimers.

## Related literature

For Schiff base compounds, see: Miura *et al.* (2009 ▶); Zhao *et al.* (2010 ▶); Karadağ *et al.* (2011) ▶; Bingöl Alpaslan *et al.* (2010 ▶). For bond-length data, see: Allen *et al.* (1987 ▶).
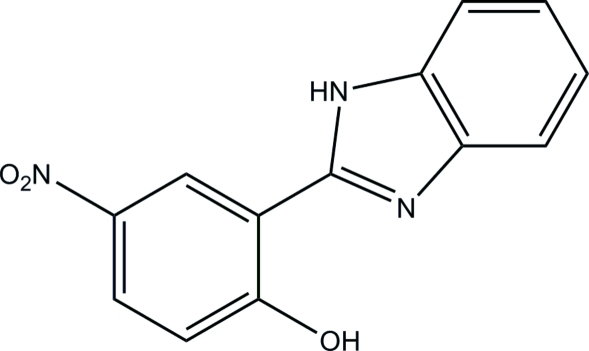


## Experimental

### 

#### Crystal data

C_13_H_9_N_3_O_3_
*M* *_r_* = 255.23
Monoclinic, 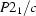

*a* = 8.117 (3) Å
*b* = 6.769 (2) Å
*c* = 20.842 (3) Å
β = 99.235 (2)°
*V* = 1130.2 (5) Å^3^
*Z* = 4
Mo *K*α radiation
μ = 0.11 mm^−1^
*T* = 298 K
0.20 × 0.20 × 0.18 mm

#### Data collection

Bruker SMART CCD area-detector diffractometer
Absorption correction: multi-scan (*SADABS*; Bruker, 2001 ▶) *T*_min_ = 0.978, *T*_max_ = 0.980
8933 measured reflections
2469 independent reflections
1283 reflections with *I* > 2σ(*I*)
*R*_int_ = 0.061

#### Refinement

*R*[*F* ^2^ > 2σ(*F* ^2^)] = 0.068
*wR*(*F* ^2^) = 0.153
*S* = 1.04
2469 reflections
176 parameters
1 restraint
H atoms treated by a mixture of independent and constrained refinement
Δρ_max_ = 0.20 e Å^−3^
Δρ_min_ = −0.14 e Å^−3^

### 

Data collection: *SMART* (Bruker, 2007 ▶); cell refinement: *SAINT* (Bruker, 2007 ▶); data reduction: *SAINT*; program(s) used to solve structure: *SHELXTL* (Sheldrick, 2008 ▶); program(s) used to refine structure: *SHELXTL*; molecular graphics: *SHELXTL*; software used to prepare material for publication: *SHELXTL*.

## Supplementary Material

Crystal structure: contains datablocks global, I. DOI: 10.1107/S1600536811001644/hg2791sup1.cif

Structure factors: contains datablocks I. DOI: 10.1107/S1600536811001644/hg2791Isup2.hkl

Additional supplementary materials:  crystallographic information; 3D view; checkCIF report

## Figures and Tables

**Table 1:** Hydrogen-bond geometry (Å, °)

*D*—H⋯*A*	*D*—H	H⋯*A*	*D*⋯*A*	*D*—H⋯*A*
N2—H2⋯O2^i^	0.90 (1)	2.02 (1)	2.898 (3)	164 (3)
O1—H1⋯N1	0.82	1.85	2.590 (3)	149

Symmetry code: (i) 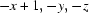.

**Fig. 1.**
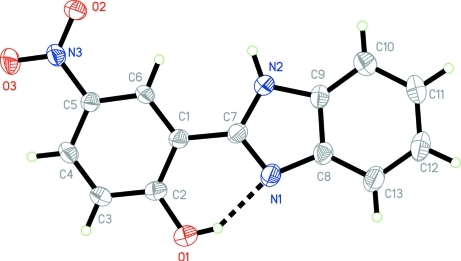
The molecular structure of the title compounds with atom labels and the 30% probability displacement ellipsoids. Intramolecular O—H···O hydrogen bond is shown as a dashed line.

**Fig. 2.**
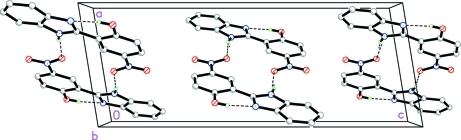
The molecular packing of the title compound. Hydrogen bonds are shown as dashed lines.

## supplementary crystallographic information

### Comment

The condensation reaction between aldehydes with organic primary amines readily forms Schiff bases containing the typical –C=N– groups (Miura *et al.*, 2009; Zhao *et al.*, 2010; Karadağ *et al.*, 2011; Bingöl Alpaslan *et al.*, 2010). In this paper, the title compound (Fig. 1) was prepared by the reaction of 5-nitrosalicylaldehyde with 1,2-diaminobenzene in methanol.

The whole molecule of the compound is approximately planar, with mean deviation from the plane defined by the non-hydrogen atoms of 0.0311 (4) Å, and with the dihedral angle between the benzene ring and the Benzimidazole ring of 1.1 (3)°. All the bond lengths are within normal ranges (Allen *et al.*, 1987). There is an intramolecular O—H···N hydrogen bond in the molecule (Table 1). In the crystal structure, adjacent two molecules are linked through intermolecular N—H···O hydrogen bonds (Table 1) to form a dimer (Fig. 2).

### Experimental

5-Nitrosalicylaldehyde (1.0 mmol, 0.167 g) and 1,2-diaminobenzene (0.5 mmol, 0.054 g) were refluxed for 30 min in 30 ml me thanol, and cooled to room temperature to give colorless solid, which was isolated by filtration. Single crystals of the title compound were formed by recrystallization of the solid in methanol.

### Refinement

H2 was located in a difference Fourier map and refined isotropically, with the N—H distance restrained to 0.90 (1) Å. The other H atoms were positioned geometrically and refined using the riding-model approximation, with C–H = 0.93 Å, and O–H = 0.82 Å, and *U*_iso_(H) = 1.2*U*_eq_(C) or *U*_iso_(H) = 1.5*U*_eq_(O).

### Crystal data

**Table:**

C_13_H_9_N_3_O_3_	*F*(000) = 528
*M**_r_* = 255.23	*D*_x_ = 1.500 Mg m^−^^3^
Monoclinic, *P*2_1_/*c*	Mo *K*α radiation, λ = 0.71073 Å
Hall symbol: -P 2ybc	Cell parameters from 1124 reflections
*a* = 8.117 (3) Å	θ = 2.5–24.5°
*b* = 6.769 (2) Å	µ = 0.11 mm^−^^1^
*c* = 20.842 (3) Å	*T* = 298 K
β = 99.235 (2)°	Block, yellow
*V* = 1130.2 (5) Å^3^	0.20 × 0.20 × 0.18 mm
*Z* = 4	

### Data collection

**Table:**

Bruker SMART CCD area-detector diffractometer	2469 independent reflections
Radiation source: fine-focus sealed tube	1283 reflections with *I* > 2σ(*I*)
graphite	*R*_int_ = 0.061
ω scans	θ_max_ = 27.0°, θ_min_ = 2.5°
Absorption correction: multi-scan (*SADABS*; Bruker, 2001)	*h* = −10→10
*T*_min_ = 0.978, *T*_max_ = 0.980	*k* = −8→8
8933 measured reflections	*l* = −26→24

### Refinement

**Table:**

Refinement on *F*^2^	Primary atom site location: structure-invariant direct methods
Least-squares matrix: full	Secondary atom site location: difference Fourier map
*R*[*F*^2^ > 2σ(*F*^2^)] = 0.068	Hydrogen site location: inferred from neighbouring sites
*wR*(*F*^2^) = 0.153	H atoms treated by a mixture of independent and constrained refinement
*S* = 1.04	*w* = 1/[σ^2^(*F*_o_^2^) + (0.0589*P*)^2^ + 0.1494*P*] where *P* = (*F*_o_^2^ + 2*F*_c_^2^)/3
2469 reflections	(Δ/σ)_max_ < 0.001
176 parameters	Δρ_max_ = 0.20 e Å^−^^3^
1 restraint	Δρ_min_ = −0.14 e Å^−^^3^

### Special details

**Table:**

Geometry. All e.s.d.'s (except the e.s.d. in the dihedral angle between two l.s. planes) are estimated using the full covariance matrix. The cell e.s.d.'s are taken into account individually in the estimation of e.s.d.'s in distances, angles and torsion angles; correlations between e.s.d.'s in cell parameters are only used when they are defined by crystal symmetry. An approximate (isotropic) treatment of cell e.s.d.'s is used for estimating e.s.d.'s involving l.s. planes.
Refinement. Refinement of *F*^2^ against ALL reflections. The weighted *R*-factor *wR* and goodness of fit *S* are based on *F*^2^, conventional *R*-factors *R* are based on *F*, with *F* set to zero for negative *F*^2^. The threshold expression of *F*^2^ > σ(*F*^2^) is used only for calculating *R*-factors(gt) *etc*. and is not relevant to the choice of reflections for refinement. *R*-factors based on *F*^2^ are statistically about twice as large as those based on *F*, and *R*- factors based on ALL data will be even larger.

### Fractional atomic coordinates and isotropic or equivalent isotropic displacement parameters (Å^2^)

**Table:**

	*x*	*y*	*z*	*U*_iso_*/*U*_eq_	
O1	0.7974 (3)	0.7492 (3)	0.12518 (10)	0.0680 (7)	
H1	0.8172	0.7776	0.0889	0.102*	
O2	0.4484 (3)	−0.0641 (3)	0.07368 (10)	0.0687 (7)	
O3	0.4555 (3)	−0.0423 (3)	0.17662 (11)	0.0764 (8)	
N1	0.8286 (3)	0.7006 (4)	0.00445 (12)	0.0547 (7)	
N2	0.7411 (3)	0.4267 (3)	−0.04913 (12)	0.0495 (7)	
N3	0.4870 (3)	0.0226 (4)	0.12571 (12)	0.0520 (7)	
C1	0.6985 (3)	0.4551 (4)	0.06634 (13)	0.0425 (7)	
C2	0.7228 (4)	0.5722 (4)	0.12303 (15)	0.0477 (8)	
C3	0.6712 (4)	0.5046 (5)	0.17926 (14)	0.0562 (9)	
H3	0.6878	0.5828	0.2164	0.067*	
C4	0.5966 (4)	0.3252 (4)	0.18085 (14)	0.0492 (8)	
H4	0.5632	0.2799	0.2189	0.059*	
C5	0.5710 (3)	0.2113 (4)	0.12517 (14)	0.0425 (7)	
C6	0.6205 (3)	0.2741 (4)	0.06832 (13)	0.0423 (7)	
H6	0.6016	0.1950	0.0314	0.051*	
C7	0.7560 (3)	0.5267 (4)	0.00778 (14)	0.0461 (7)	
C8	0.8628 (4)	0.7151 (4)	−0.05876 (14)	0.0484 (8)	
C9	0.8092 (3)	0.5440 (4)	−0.09285 (15)	0.0475 (7)	
C10	0.8259 (4)	0.5165 (5)	−0.15689 (15)	0.0594 (9)	
H10	0.7902	0.4011	−0.1791	0.071*	
C11	0.8983 (4)	0.6692 (5)	−0.18671 (16)	0.0645 (10)	
H11	0.9114	0.6565	−0.2300	0.077*	
C12	0.9518 (4)	0.8413 (5)	−0.15324 (18)	0.0694 (10)	
H12	0.9996	0.9414	−0.1747	0.083*	
C13	0.9354 (4)	0.8666 (5)	−0.08904 (17)	0.0645 (9)	
H13	0.9719	0.9816	−0.0668	0.077*	
H2	0.698 (4)	0.307 (2)	−0.0610 (15)	0.080*	

### Atomic displacement parameters (Å^2^)

**Table:**

	*U*^11^	*U*^22^	*U*^33^	*U*^12^	*U*^13^	*U*^23^
O1	0.0862 (18)	0.0561 (14)	0.0638 (17)	−0.0234 (12)	0.0183 (14)	−0.0113 (11)
O2	0.1090 (19)	0.0552 (13)	0.0445 (14)	−0.0268 (12)	0.0203 (13)	−0.0100 (11)
O3	0.122 (2)	0.0662 (15)	0.0487 (14)	−0.0165 (14)	0.0369 (14)	0.0101 (12)
N1	0.0620 (18)	0.0552 (15)	0.0460 (17)	−0.0161 (13)	0.0055 (13)	0.0067 (13)
N2	0.0588 (17)	0.0435 (14)	0.0465 (16)	−0.0102 (12)	0.0096 (13)	0.0012 (13)
N3	0.0713 (18)	0.0478 (15)	0.0403 (16)	−0.0029 (13)	0.0192 (13)	0.0019 (13)
C1	0.0475 (18)	0.0399 (16)	0.0387 (17)	−0.0038 (14)	0.0033 (14)	0.0005 (13)
C2	0.0458 (18)	0.0434 (17)	0.052 (2)	−0.0057 (14)	0.0037 (15)	−0.0061 (15)
C3	0.070 (2)	0.058 (2)	0.0413 (19)	−0.0124 (17)	0.0117 (16)	−0.0126 (16)
C4	0.061 (2)	0.0553 (19)	0.0328 (17)	−0.0008 (16)	0.0108 (15)	−0.0021 (15)
C5	0.0489 (18)	0.0390 (15)	0.0393 (18)	−0.0027 (14)	0.0058 (14)	0.0026 (13)
C6	0.0547 (18)	0.0418 (16)	0.0302 (16)	−0.0045 (14)	0.0061 (13)	−0.0025 (13)
C7	0.0497 (18)	0.0412 (16)	0.0462 (19)	−0.0073 (14)	0.0038 (14)	0.0028 (15)
C8	0.0494 (19)	0.0516 (18)	0.0422 (19)	−0.0055 (15)	0.0014 (15)	0.0085 (15)
C9	0.0442 (18)	0.0535 (18)	0.0447 (19)	−0.0026 (15)	0.0070 (14)	0.0107 (15)
C10	0.063 (2)	0.065 (2)	0.050 (2)	0.0030 (17)	0.0071 (16)	0.0009 (17)
C11	0.069 (2)	0.082 (3)	0.045 (2)	0.003 (2)	0.0157 (18)	0.0130 (19)
C12	0.070 (2)	0.075 (2)	0.064 (3)	−0.009 (2)	0.0121 (19)	0.028 (2)
C13	0.070 (2)	0.061 (2)	0.062 (2)	−0.0165 (18)	0.0115 (18)	0.0127 (18)

### Geometric parameters (Å, °)

**Table:**

O1—C2	1.340 (3)	C3—H3	0.9300
O1—H1	0.8200	C4—C5	1.381 (4)
O2—N3	1.228 (3)	C4—H4	0.9300
O3—N3	1.213 (3)	C5—C6	1.378 (4)
N1—C7	1.323 (3)	C6—H6	0.9300
N1—C8	1.393 (4)	C8—C13	1.384 (4)
N2—C7	1.354 (3)	C8—C9	1.392 (4)
N2—C9	1.388 (3)	C9—C10	1.376 (4)
N2—H2	0.902 (10)	C10—C11	1.385 (4)
N3—C5	1.449 (3)	C10—H10	0.9300
C1—C6	1.383 (4)	C11—C12	1.391 (5)
C1—C2	1.410 (4)	C11—H11	0.9300
C1—C7	1.458 (4)	C12—C13	1.376 (5)
C2—C3	1.384 (4)	C12—H12	0.9300
C3—C4	1.360 (4)	C13—H13	0.9300
			
C2—O1—H1	109.5	C5—C6—H6	120.1
C7—N1—C8	105.7 (2)	C1—C6—H6	120.1
C7—N2—C9	107.5 (2)	N1—C7—N2	112.1 (2)
C7—N2—H2	132 (2)	N1—C7—C1	123.0 (3)
C9—N2—H2	121 (2)	N2—C7—C1	124.9 (2)
O3—N3—O2	122.7 (3)	C13—C8—C9	120.3 (3)
O3—N3—C5	119.4 (3)	C13—C8—N1	130.4 (3)
O2—N3—C5	117.9 (2)	C9—C8—N1	109.3 (2)
C6—C1—C2	118.4 (3)	C10—C9—N2	132.1 (3)
C6—C1—C7	121.9 (2)	C10—C9—C8	122.5 (3)
C2—C1—C7	119.6 (2)	N2—C9—C8	105.4 (3)
O1—C2—C3	117.6 (3)	C9—C10—C11	116.7 (3)
O1—C2—C1	122.2 (3)	C9—C10—H10	121.7
C3—C2—C1	120.2 (3)	C11—C10—H10	121.7
C4—C3—C2	120.8 (3)	C10—C11—C12	121.4 (3)
C4—C3—H3	119.6	C10—C11—H11	119.3
C2—C3—H3	119.6	C12—C11—H11	119.3
C3—C4—C5	119.1 (3)	C13—C12—C11	121.4 (3)
C3—C4—H4	120.5	C13—C12—H12	119.3
C5—C4—H4	120.5	C11—C12—H12	119.3
C6—C5—C4	121.7 (3)	C12—C13—C8	117.7 (3)
C6—C5—N3	118.8 (2)	C12—C13—H13	121.1
C4—C5—N3	119.6 (3)	C8—C13—H13	121.1
C5—C6—C1	119.8 (2)		

### Hydrogen-bond geometry (Å, °)

**Table:**

*D*—H···*A*	*D*—H	H···*A*	*D*···*A*	*D*—H···*A*
N2—H2···O2^i^	0.90 (1)	2.02 (1)	2.898 (3)	164 (3)
O1—H1···N1	0.82	1.85	2.590 (3)	149

Symmetry codes: (i) −*x*+1, −*y*, −*z*.
